# Behavioral and Biochemical Effects of an Arylhydrazone Derivative of 5-Methoxyindole-2-Carboxylic Acid in a Scopolamine-Induced Model of Alzheimer’s Type Dementia in Rats

**DOI:** 10.3390/molecules29235711

**Published:** 2024-12-03

**Authors:** Polina Petkova-Kirova, Neda Anastassova, Borislav Minchev, Diamara Uzunova, Valya Grigorova, Elina Tsvetanova, Almira Georgieva, Albena Alexandrova, Miroslava Stefanova, Denitsa Yancheva, Reni Kalfin, Lyubka Tancheva

**Affiliations:** 1 Institute of Neurobiology, Bulgarian Academy of Sciences, Acad. G. Bonchev Str., bl. 23, 1113 Sofia, Bulgaria; p.kirova@inb.bas.bg (P.P.-K.); boby_al@mail.bg (B.M.); didi_uzunova1@abv.bg (D.U.); vbgrigorova@abv.bg (V.G.); elina_nesta@abv.bg (E.T.); a.georgieva@inb.bas.bg (A.G.); a.alexandrova@inb.bas.bg (A.A.); mira_stefanova@mail.bg (M.S.); reni_kalfin@abv.bg (R.K.); 2 Institute of Organic Chemistry with Centre of Phytochemistry, Bulgarian Academy of Sciences, Acad. G. Bonchev Str., Building 9, 1113 Sofia, Bulgaria; neda.anastassova@orgchm.bas.bg; 3 Department of Organic Chemistry, University of Chemical Technology and Metallurgy, 8 Kliment Ohridski Blvd., 1756 Sofia, Bulgaria; 4 Department of Physiology and Biochemistry, National Sports Academy, Acad. S. Mladenov Str. 21, 1700 Sofia, Bulgaria; 5 Department of Healthcare, Faculty of Public Health, Healthcare and Sport, South-West University, Ivan Mihailov 66, 2700 Blagoevgrad, Bulgaria

**Keywords:** Alzheimer’s disease, arylhydrazone derivatives of 5-methoxyindole-2-carboxylic acid, scopolamine, step-through, Barnes maze, acetylcholine, acetylcholine esterase, BDNF, lipid peroxidation, catalase

## Abstract

Alzheimer’s disease (AD) has long proven to be a complex neurodegenerative disorder, with cholinergic dysfunction, oxidative stress, and neuroinflammation being just a few of its pathological features. The complexity of the disease requires a multitargeted treatment covering its many aspects. In the present investigation, an arylhydrazone derivative of 5-methoxyindole-2-carboxylic acid (5MeO), with in vitro strong antioxidant, neuroprotective and monoamine oxidase B-inhibiting effects, was studied in a scopolamine-induced Alzheimer-type dementia in rats. Using behavioral and biochemical methods, we evaluated the effects of 5MeO on learning and memory, and elucidated the mechanisms of these effects. Our experiments demonstrated that 5MeO had a beneficial effect on different types of memory as assessed by the step-through and the Barnes maze tasks. It efficiently restored the decreased by scopolamine brain-derived neurotrophic factor and acetylcholine levels and normalized the increased by scopolamine acetylcholine esterase activity in hippocampus. Most effective 5MeO was in counteracting the induced by scopolamine oxidative stress by decreasing the increased by scopolamine levels of lipid peroxidation and by increasing the reduced by scopolamine catalase activity. Blood biochemical analyses demonstrated a favorable safety profile of 5MeO, prompting further pharmacological studies suggesting 5MeO as a safe and efficient candidate in a multitargeted treatment of AD.

## 1. Introduction

Alzheimer’s disease (AD) represents a significant global health challenge, as it is the most prevalent neurodegenerative disorder, currently affecting over 55 million people worldwide. The chronic and progressive disease is hallmarked by acetylcholine deficiency, the accumulation of amyloid-β (Aβ) plaques, and hyperphosphorylated tau protein aggregates forming neurofibrillary tangles (NFT), which trigger oxidative stress, gradual memory decline, and cognitive impairment that profoundly affect both personality and social functioning [1]. In recent years, mortality rates attributable to AD have reached levels comparable to those of cardiovascular diseases and cancer [2]. Estimates indicate that the global incidence of AD will rise exponentially, with cases expected to double by 2040, underscoring the urgent need for the development of effective treatment and therapeutic interventions [3].

Despite significant advances in the understanding of AD, its precise etiology remains elusive. Multiple hypotheses have been proposed to explain its pathological onset, most notably cholinergic degeneration, amyloid-β (Aβ) aggregation, and tau hyperphosphorylation [4]. However, these mechanisms are now recognized as key components of a broader, multifactorial process. Genetic predispositions, such as mutations in APP, PSEN1, and PSEN2, along with environmental factors like lifestyle and comorbidities, play key roles. Additionally, neuroinflammation, oxidative stress, mitochondrial dysfunction, and impaired autophagy are critical contributors to AD progression [5,6].

Current therapies primarily target the cholinergic system with acetylcholinesterase inhibitors, such as donepezil, rivastigmine, and galantamine, or modulate the glutamatergic system with N-methyl-D-aspartate (NMDA) receptor antagonists like memantine [7]. While those treatments provide symptomatic relief, they do not alter disease progression and often cause adverse effects, such as gastrointestinal and cardiovascular complications. The 2021, nearly two decades after the approval of memantine, FDA approval of aducanumab, the first monoclonal antibody targeting Aβ, offers new hopes but also raises concerns related to its high cost, clinical efficacy, and overall benefits [8].

Given the multifactorial nature of AD, there is growing consensus that multi-targeted therapeutic approaches, which simultaneously address Aβ and tau pathology, neuroinflammation, and oxidative stress, are crucial for advancing disease management [9,10,11]. The exploration of novel complex treatment strategies seems increasingly important.

The trigger of AD pathogenesis remains unknown, but evidence has emerged regarding early molecular events. Post-mortem analyses reveal increased peroxidation of lipids, proteins, and DNA in AD brains, with oxidative damage preceding Aβ plaque formation, indicating its upstream role in neurodegeneration [12,13,14,15,16]. Reactive oxygen species (ROS) generated by mitochondrial dysfunction and metal ion dysregulation, particularly of iron and copper, exacerbate synaptic dysfunction and neuronal damage, fostering Aβ accumulation and tau hyperphosphorylation. The implication of oxidative stress as an early pathological event highlights the importance of exploring antioxidant-based and neuroprotective therapies. Although current antioxidant approaches have shown limited clinical success, targeting oxidative stress alongside other mechanisms may offer more effective strategies [17,18].

Elevated monoamine oxidase-B (MAO-B) levels in AD contribute to increased ROS production and Aβ formation, linking oxidative stress with amyloid pathology [19,20]. This dual role makes MAO-B a key target in AD progression. MAO-B inhibitors have garnered significant attention as potential therapeutic agents. By inhibiting MAO-B, it is possible to reduce the oxidative burden and the production of Aβ peptides, offering a multi-targeted approach. Although MAO-B inhibitors such as selegiline have shown some promise in preclinical studies, their clinical efficacy in AD remains under investigation. Nonetheless, combining MAO-B inhibition with other therapeutic strategies may enhance neuroprotection and slow AD progression, highlighting its therapeutic potential.

In our previous research, we investigated benzimidazole and indole-arylhydrazone hybrids as potential anti-Parkinsonian multi-target drugs, which revealed that the presence of the arylhydrazone moiety provided significant advantages beyond typical neuroprotective activity [21,22,23]. This structural feature, in combination with hydroxy and methoxy substituents, led to MAO-B inhibition, alongside enhanced neuroprotective and antioxidant properties. Notably, the in vitro experiments demonstrated that the neuroprotective effects of these hybrids surpassed those of melatonin and the clinically established Parkinson’s disease treatment rasagiline. These findings suggest that arylhydrazone derivatives represent a promising class of multi-functional therapeutic agents.

**Scheme 1:**
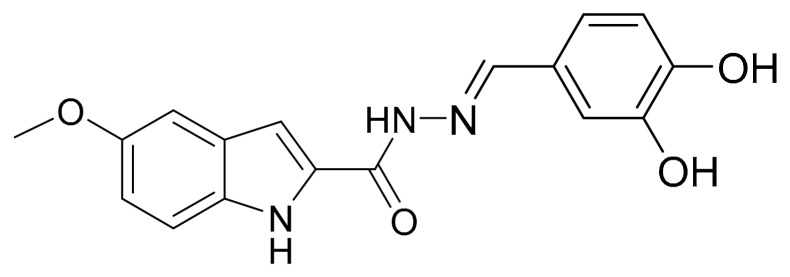
Molecular structure of the target compound N′-(3,4-dihydroxybenzylidene)-5-methoxy-1H-indole-2-carbohydrazide (5MeO).

Among the arylhydrazone derivatives of 5-methoxyindole-2-carboxylic acid, the compound containing a 3,4-dihydroxy functional group (5MeO; see Scheme 1) was extensively studied in various assays [22]. It exhibited a favorable safety profile and potent neuroprotective effects, outperforming reference compounds such as indole-3-propionic acid (IPA), melatonin, and rasagiline, as well as a prominent antioxidant capacity suppressing lipid peroxidation and deoxyribose degradation, with a higher potency than melatonin and Trolox [24,25,26]. Furthermore, 5MeO significantly inhibited human recombinant MAO-B activity, supported by molecular docking studies confirming its optimal fit into the enzyme’s flat hydrophobic cavity. Its ability to cross the blood–brain barrier further supports 5MeO as a compelling candidate for in vivo AD studies.

The aim of the present study was to evaluate the effects of 5MeO in a scopolamine model of Alzheimer-type dementia in rats by using behavioral and biochemical methods.

## 2. Results

### 2.1. Behavioral Assessment of the Effects of 5MeO on Learning and Memory

#### 2.1.1. Step-Through Inhibitory Avoidance

**Figure 1:**
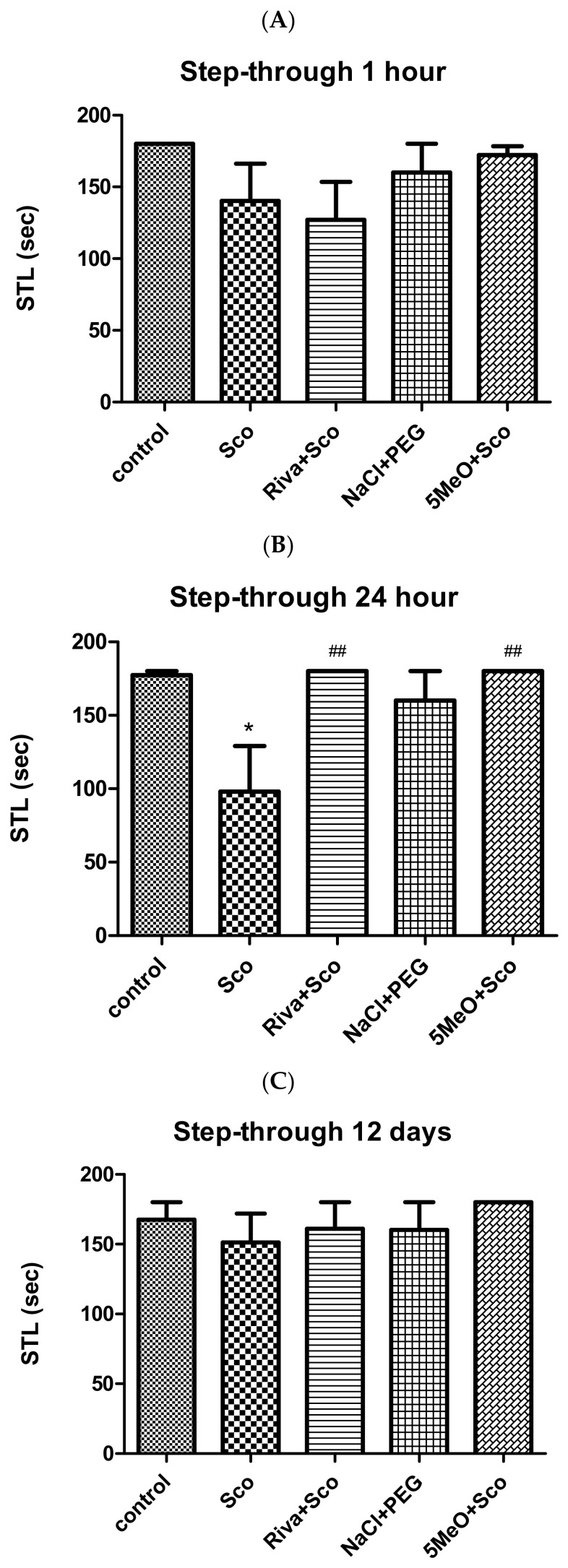
Effects of 5MeO (14.7 μM) on learning and memory in a scopolamine model of dementia in rats. Step-through latency is assessed at 1 h, 24 h, and on the 12th day ((**A**), (**B**), and (**C**), respectively) after the beginning of scopolamine treatment ((**A**), (**B**), and (**C**), respectively) for the control, Sco, Riva + Sco, 5MeO + Sco, and NaCl + PEG group. Data are expressed as mean ± SEM; significant differences among experimental groups are denoted with “*” when the differences are with the saline (control) group and denoted with “#” when the differences are with the dementia (Sco) group. The number of “*” or “#” above bars is assigned as follows: * at *p* < 0.05; ## at *p* < 0.01.

In Figure 1A–C, it is shown that scopolamine treatment significantly decreases the step-through latency (STL) at 24 h without having a significant effect on the 1st hour and on the 12th day after the beginning of scopolamine treatment. At 24 h, all three groups (Riva + Sco; 5MeO + Sco; NaCl + PEG) demonstrated no significant differences compared to the control group; however, the groups treated with 5MeO and rivastigmine in the presence of scopolamine showed a significant difference compared to the scopolamine-treated group and STL values completely restored to those of the control group.

#### 2.1.2. Barnes Maze

**Figure 2:**
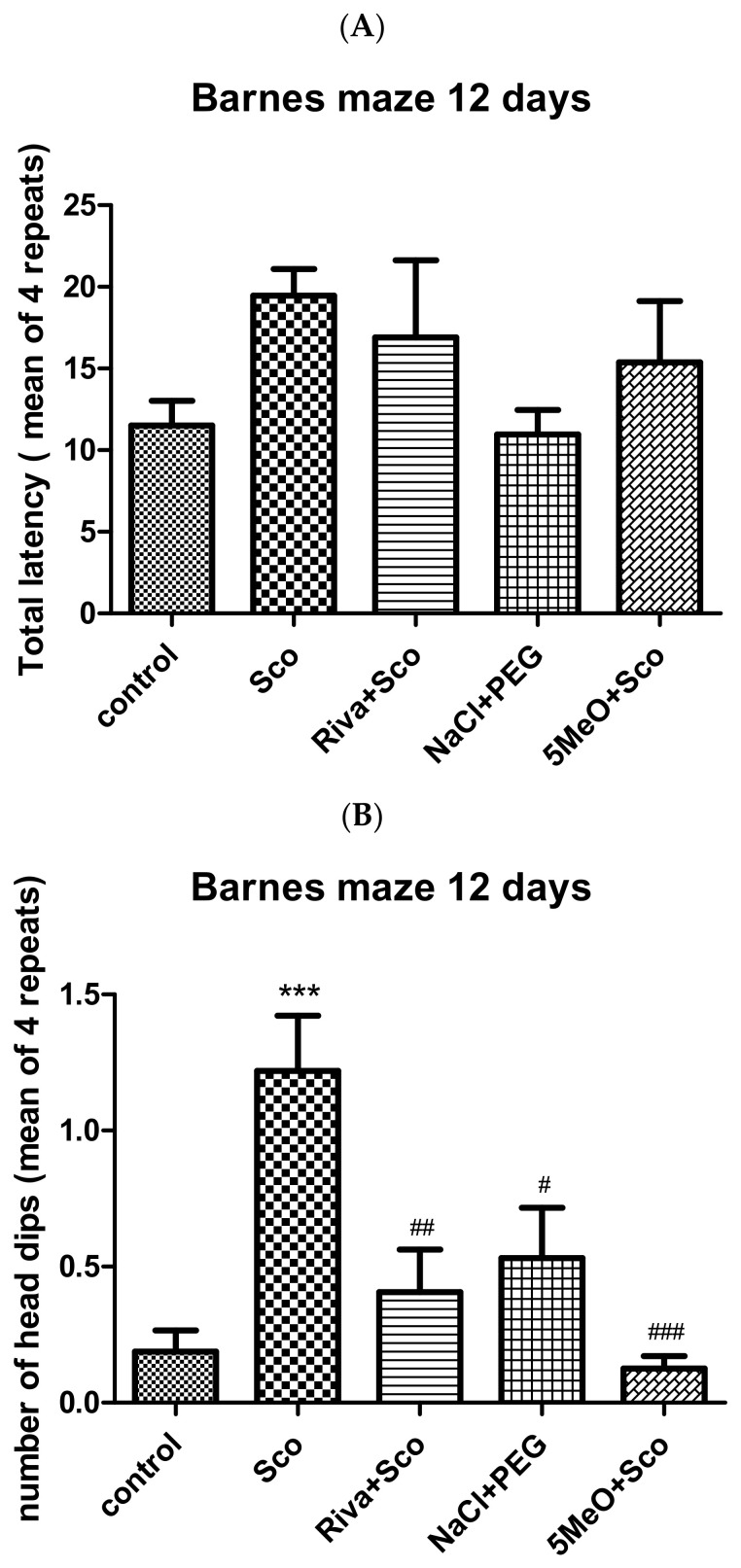
Effects of 5MeO (14.7 μM) on learning and memory in a scopolamine model of dementia in rats. Barnes maze total latency (**A**) and number of head dips (**B**) are assessed when Barnes maze training was performed under scopolamine treatment for the control, Sco, Riva + Sco, 5MeO + Sco, and NaCl + PEG groups. Data are expressed as mean ± SEM; significant differences among experimental groups are denoted with “*” when the differences are with the saline (control) group and denoted with “#” when the differences are with the dementia (Sco) group. The number of “*” or “#” above bars is assigned as follows: *** at *p* < 0.001; # at *p* < 0.05, ## at *p* < 0.01, ### at *p* < 0.001.

Figure 2 shows the effect on the Barnes maze performance, total latency, and number of head dips of 5MeO and rivastigmine (in the presence of Sco) as well as of PEG (when the Barnes maze training was carried out under scopolamine treatment). While there was no significant difference in the total latency between any of the test groups and the control group, scopolamine significantly (control vs. Sco, *p* < 0.001) increased the number of holes that the animals examined before finding the hole with the escape box underneath. Both rivastigmine and 5 MeO restored the control values although 5MeO proved more efficient than rivastigmine (Sco vs. Riva + Sco, *p* < 0.01;Sco vs. 5MeO + Sco, *p* < 0.001).

### 2.2. Biochemical Assessment of the Effects of 5MeO

#### 2.2.1. Effect of 5MeO on Brain AChE Activity

**Figure 3:**
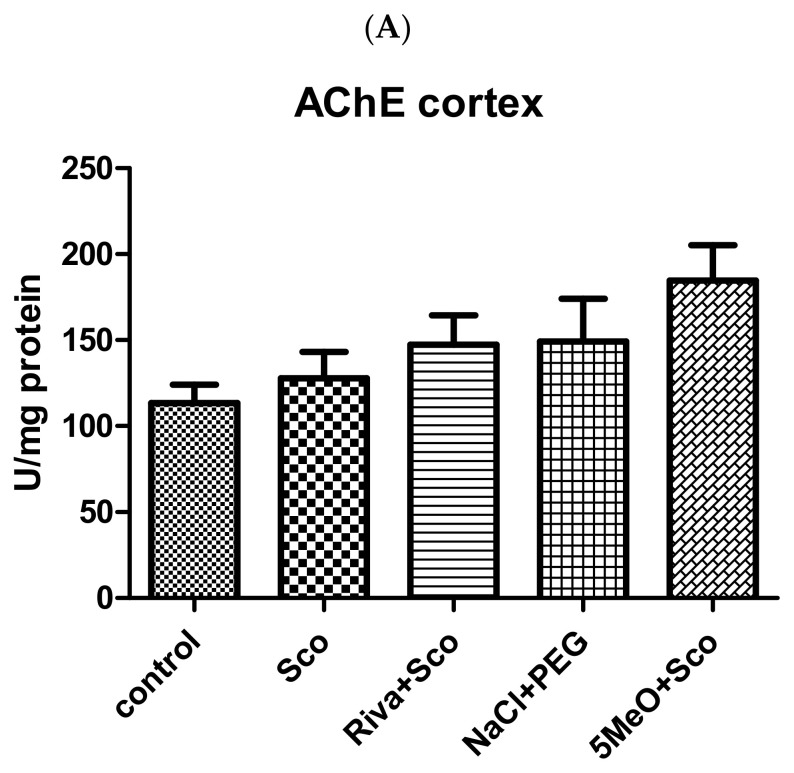

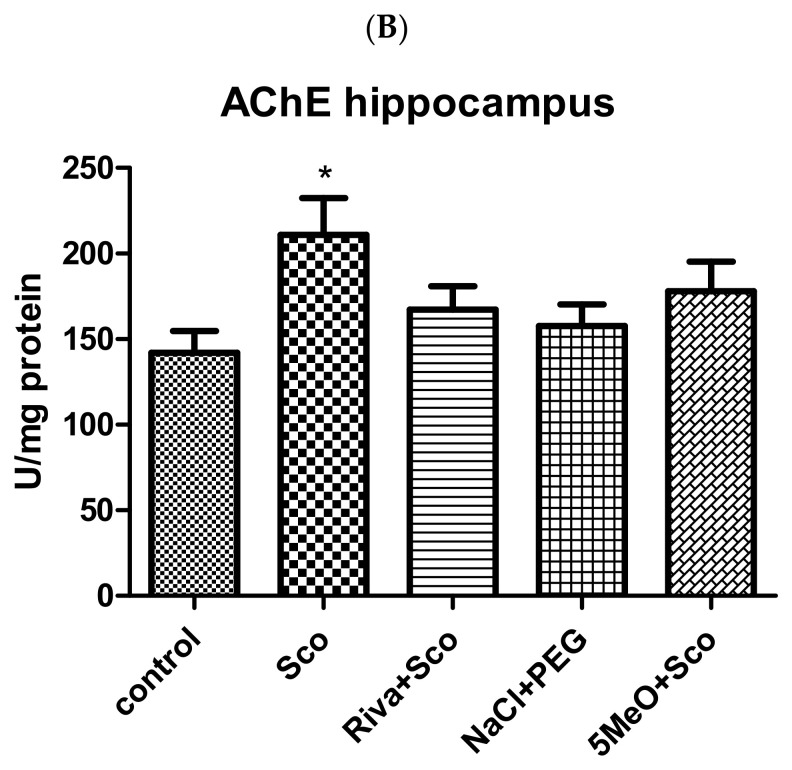
Effects of 5MeO (14.7 μM) on AChE in cortex (**A**) and hippocampus (**B**) of scopolamine-treated rats. Data are expressed as mean ± SEM; significant differences among experimental groups are denoted with “*” when the differences are with the saline (control) group and denoted with “#” when the differences are with the dementia (Sco) group. The number of “*” or “#” above bars is assigned as follows: * at *p* < 0.05.

Figure 3 shows that, while, in the cortex, scopolamine did not enhance AChE activity, in the hippocampus, it induced a significant increase (control vs. Sco, *p* < 0.05). In the cortex, no significant difference was observed among any of the examined groups. In the hippocampus, both 5MeO and rivastigmine decreased the scopolamine-induced AChE activity increase and no difference was observed between the control group of rats and the rats treated with 5MeO and scopolamine (control vs. 5MeO + Sco, *p* > 0.05) nor between the control group and the group treated with rivastigmine and scopolamine (control vs. Riva + Sco, *p* > 0.05). The rats treated with PEG also did not show any difference in AChE activity compared to the control group.

#### 2.2.2. Effect of 5MeO on Brain ACh

**Figure 4:**
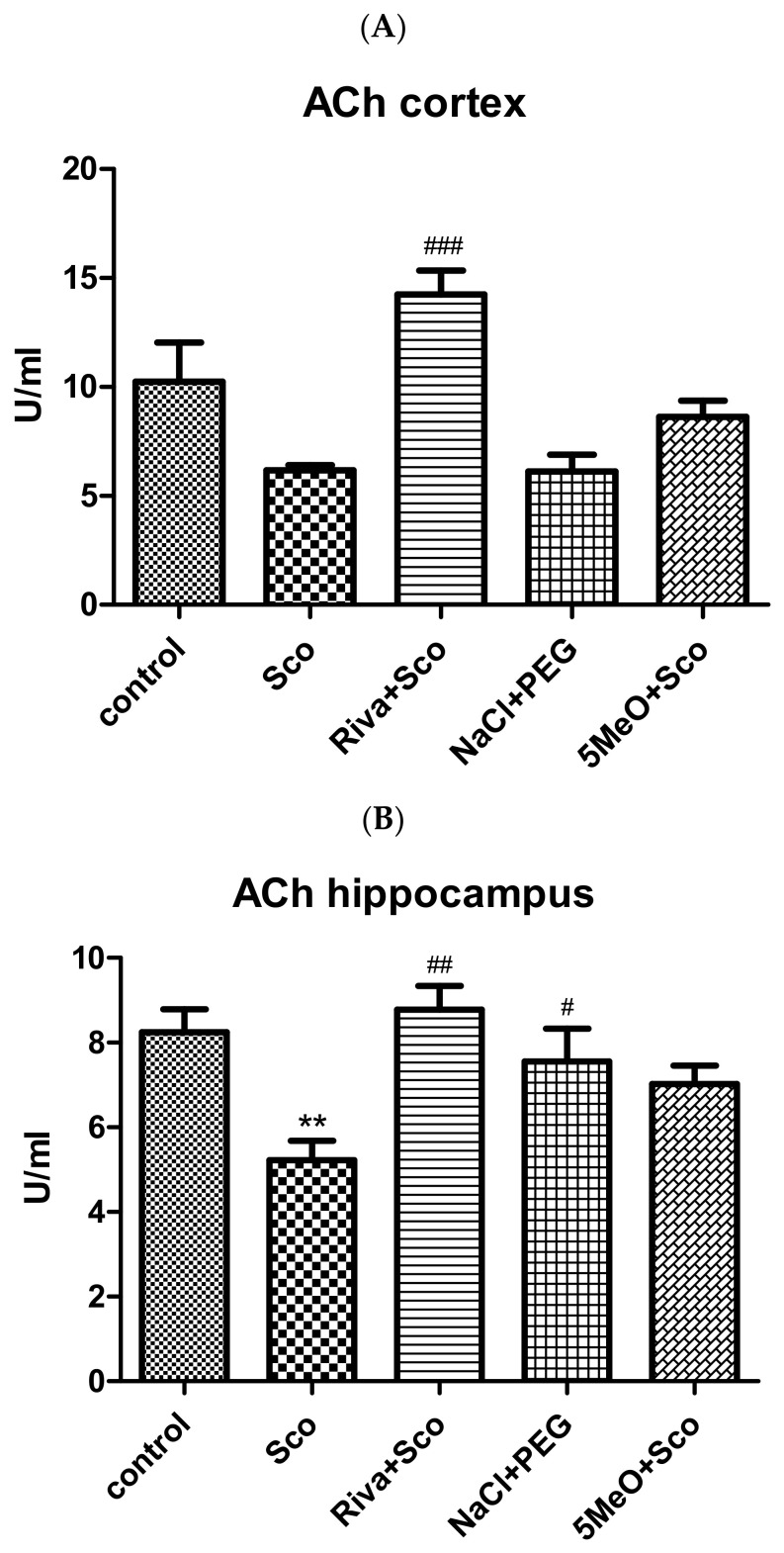
Effects of 5MeO (14.7 μM) on ACh in cortex (**A**) and hippocampus (**B**) of scopolamine-treated rats. Data are expressed as mean ± SEM; significant differences among experimental groups are denoted with “*” when the differences are with the saline (control) group and denoted with “#” when the differences are with the dementia (Sco) group. The number of “*” or “#” above bars is assigned as follows: # at *p* < 0.05; ## or ** at *p* < 0.01; ### at *p* < 0.001.

Figure 4 shows that, while, in the cortex, scopolamine did not significantly reduce the levels of ACh compared to controls, in the hippocampus, it induced a significant decrease (control vs. Sco, *p* < 0.01). In the cortex, there was no significant difference between the control group and any of the three other groups (Riva + Sco; 5MeO + Sco; NaCl + PEG) either; however, the scopolamine-treated group showed a significant difference compared to the rivastigmine and scopolamine-treated group (Sco vs. Riva + Sco, *p* < 0.001) (and the levels of ACh in the Riva + Sco injected animals were the highest). In the hippocampus, both 5MeO and rivastigmine counteracted the decrease in ACh levels induced by scopolamine and no difference between the control and Riva + Sco group nor between the control and 5MeO + Sco group was observed. The rats treated with PEG also did not show any difference in ACh levels compared to the control group. A significant increase in Ach levels, however, was observed in the rivastigmine and PEG groups compared to the scopolamine group (Sco vs. Riva + Sco, *p* < 0.01; Sco vs. NaCl + PEG, *p* < 0.05).

#### 2.2.3. Antioxidant Potential of 5MeO

##### Effect of 5MeO on Brain Levels of MDA as a Measure of Lipid Peroxidation (LPO)

**Figure 5:**
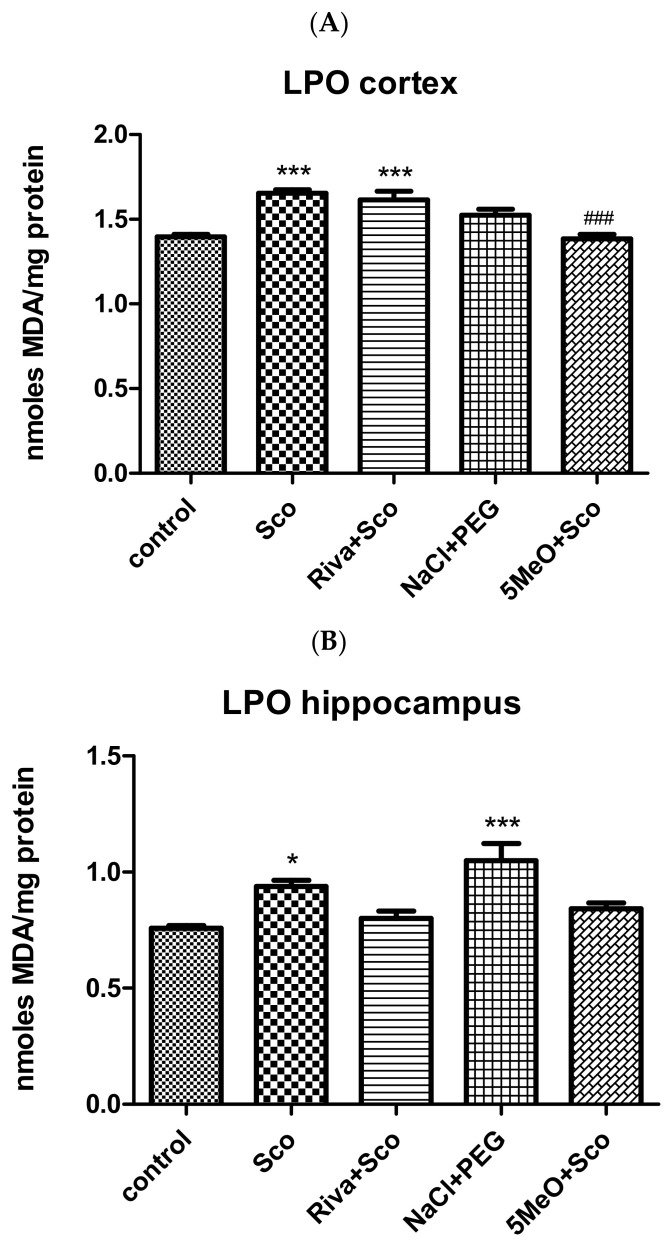
Effects of 5MeO (14.7 μM) on the MDA levels (as a measure of lipid peroxidation, LPO) in cortex (**A**) and hippocampus (**B**) of scopolamine-treated rats. Data are expressed as mean ± SEM; significant differences among experimental groups are denoted with “*” when the differences are with the saline (control) group and denoted with “#” when the differences are with the dementia (Sco) group. The number of “*” or “#” above bars is assigned as follows: * at *p* < 0.05; ### or *** at *p* < 0.001.

The levels of MDA, and thus, LPO, were significantly increased by scopolamine in both the cortex and hippocampus (Figure 5). In the cortex, rivastigmine failed to return the levels of MDA significantly increased by scopolamine to the control values, and thus, there was a significant difference between the control and the Riva + Sco group (control vs. Riva + Sco, *p* < 0.001). In turn, 5MeO returned the levels of MDA to control values and, thus, there was no difference in the MDA levels between the control and the 5MeO + Sco group (control vs. 5MeO + Sco, *p* > 0.05) but a significant decrease in the MDA levels in the 5MeO + Sco group compared to the scopolamine group (Sco vs. 5MeO + Sco, *p* < 0.001). Interestingly, a significant difference is observed also between the Riva + Sco and 5MeO + Sco groups (Riva + Sco vs. 5MeO + Sco, *p* < 0.001), showing the much better ability of 5MeO, compared to rivastigmine, to counteract the scopolamine-induced LPO. In the hippocampus, in the groups 5MeO + Sco and Riva + Sco, the MDA levels were restored to control levels and both groups showed no significant difference compared to the control group. However, the control group showed a difference compared to the PEG-treated group, in which the MDA levels were significantly increased (control vs. NaCl +PEG, *p* < 0.001).

##### Effect of 5MeO on SOD, Catalase, and GPx Activity

**Figure 6:**
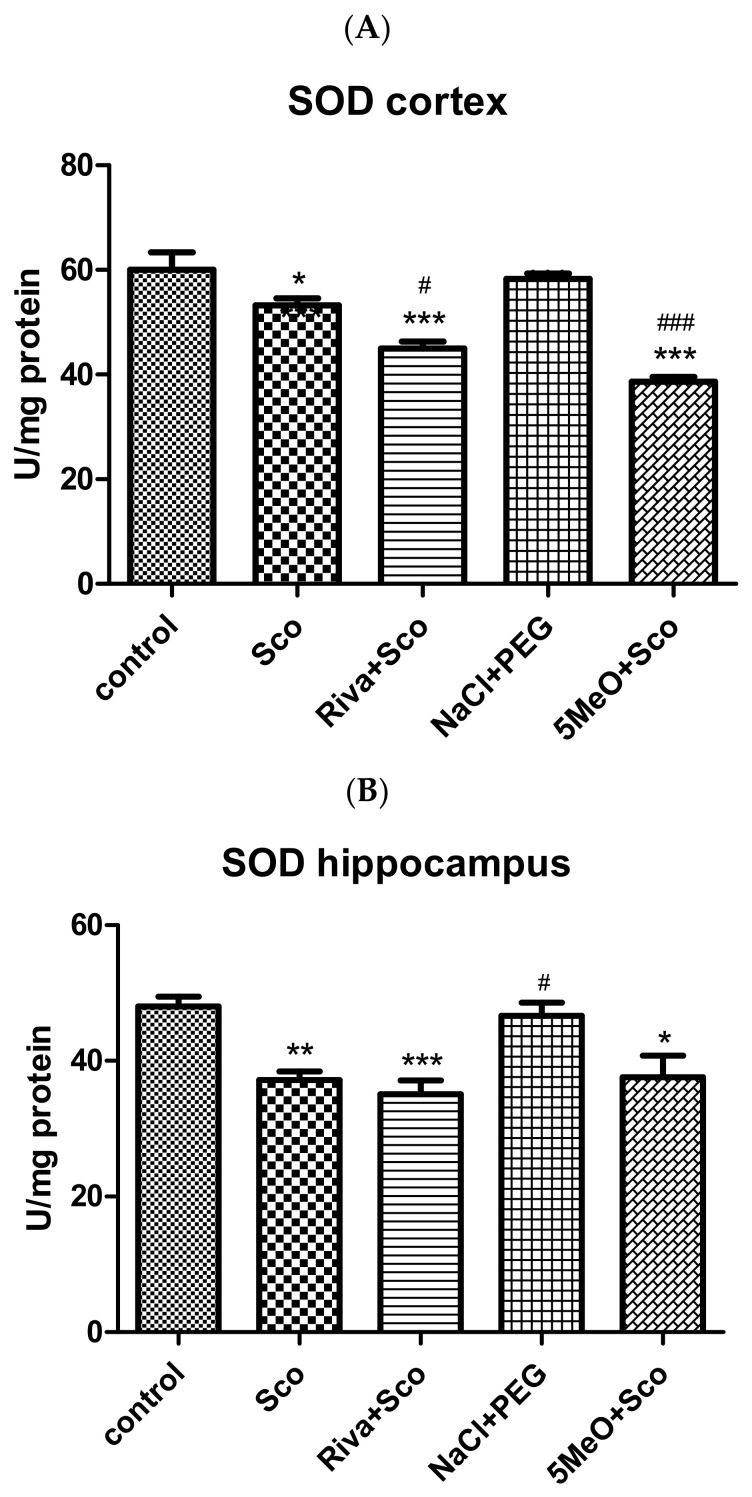

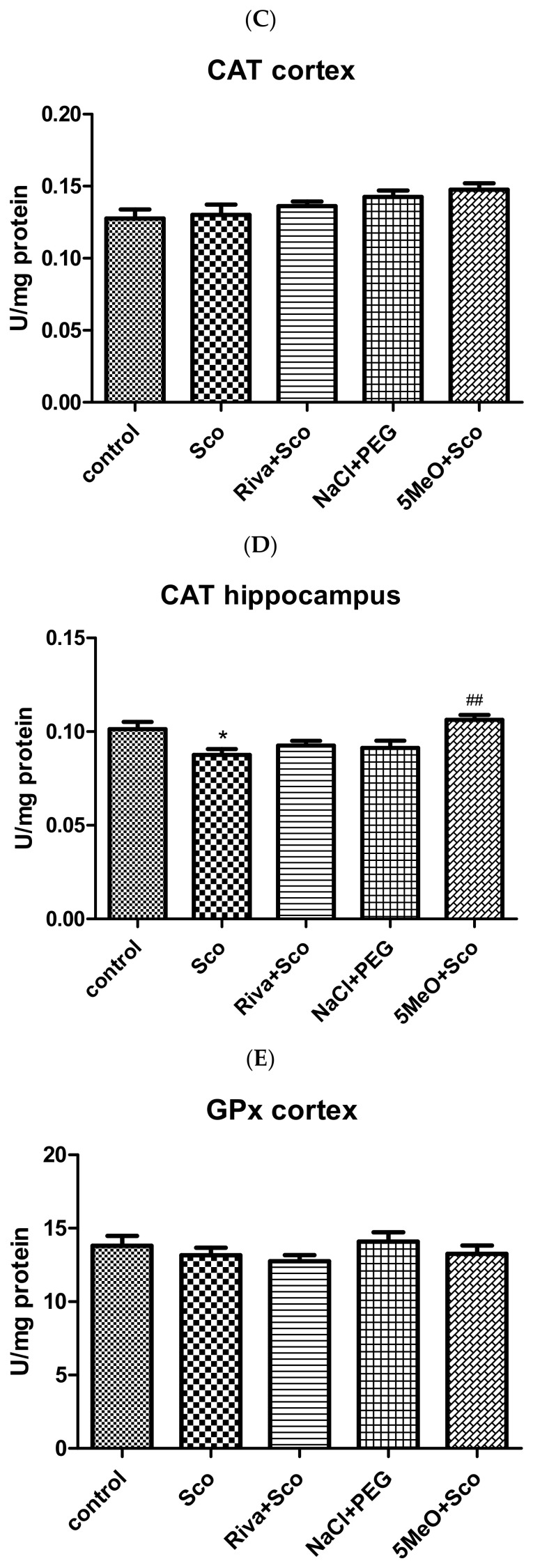

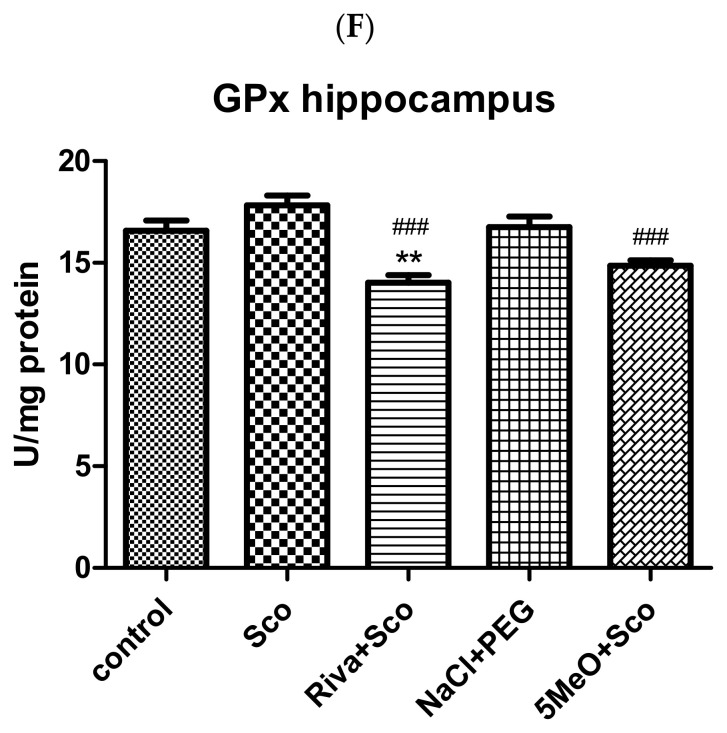
Effects of 5MeO (14.7 μM) on SOD, catalase, and GPx activity in cortex (**A**,**C**,**E**) and on SOD, catalase and GPx activity in hippocampus (**B**,**D**,**F**) of scopolamine-treated rats. Data are expressed as mean ± SEM; significant differences among experimental groups are denoted with “*” when the differences are with the saline (control) group and denoted with “#” when the differences are with the dementia (Sco) group. The number of “*” or “#” above bars is assigned as follows: # or * at *p* < 0.05; ## or ** at *p* < 0.01; ### or *** at *p* < 0.001.

SOD activity was significantly decreased by scopolamine in both the cortex and hippocampus (control vs. Sco, *p* < 0.05 cortex; control vs. Sco, *p* < 0.01 hippocampus; Figure 6A,B). In the cortex, a significant decrease in SOD activity, compared to the control group, was also observed for the Riva + Sco and 5MeO + Sco groups (control vs. Riva + Sco, *p* < 0.001; control vs. 5MeO + Sco, *p* < 0.001). A significant decrease in SOD activity for the Riva + Sco and 5MeO + Sco groups was observed also compared to the scopolamine group (Sco vs. Riva + Sco, *p* < 0.05; Sco vs. 5MeO + Sco, *p* < 0.001). Similarly, in the hippocampus, the Riva + Sco and 5MeO + Sco groups showed a decreased SOD activity compared to the control group (Sco vs. Riva + Sco, *p* < 0.001; Sco vs. 5MeO + Sco, *p* < 0.05). An increase in SOD activity was registered in the NaCl + PEG group compared to the scopolamine group (scopolamine vs. NaCl + PEG, *p* < 0.5).

Figure 6C,D shows that while, in the cortex, scopolamine did not significantly reduce CAT activity compared to controls, in the hippocampus, it induced a significant decrease (control vs. Sco, *p* < 0.05). In the cortex, there was no significant difference between the control or scopolamine group and any of the three other groups (Riva + Sco; 5MeO + Sco; NaCl + PEG). In the hippocampus, no group (except the scopolamine group) showed a significant decrease in CAT activity compared to the control group. The best efficiency in restoring CAT activity was demonstrated by 5MeO, in the presence of which the scopolamine-injected animals showed a significant increase in CAT activity compared to the group treated only with scopolamine (Sco vs. 5Meo + Sco, *p* < 0.01).

In both the cortex and hippocampus, no significant differences in GPx activity were measured between the control and scopolamine-treated groups (Figure 6E,F). In the cortex, there was no significant difference between the control or scopolamine group and any of the three other groups (Riva + Sco; 5MeO + Sco; NaCl + PEG). In the hippocampus, the only group that showed a significant decrease in GPx activity compared to the control group was the Riva + Sco group. The Riva + Sco group, but also the 5MeO + Sco group, showed a significant difference compared to the scopolamine group as well (Sco vs. Riva + Sco, *p* < 0.001; Sco vs. 5MeO + Sco, *p* < 0.001)

#### 2.2.4. Effect of 5MeO on Brain BDNF

**Figure 7:**
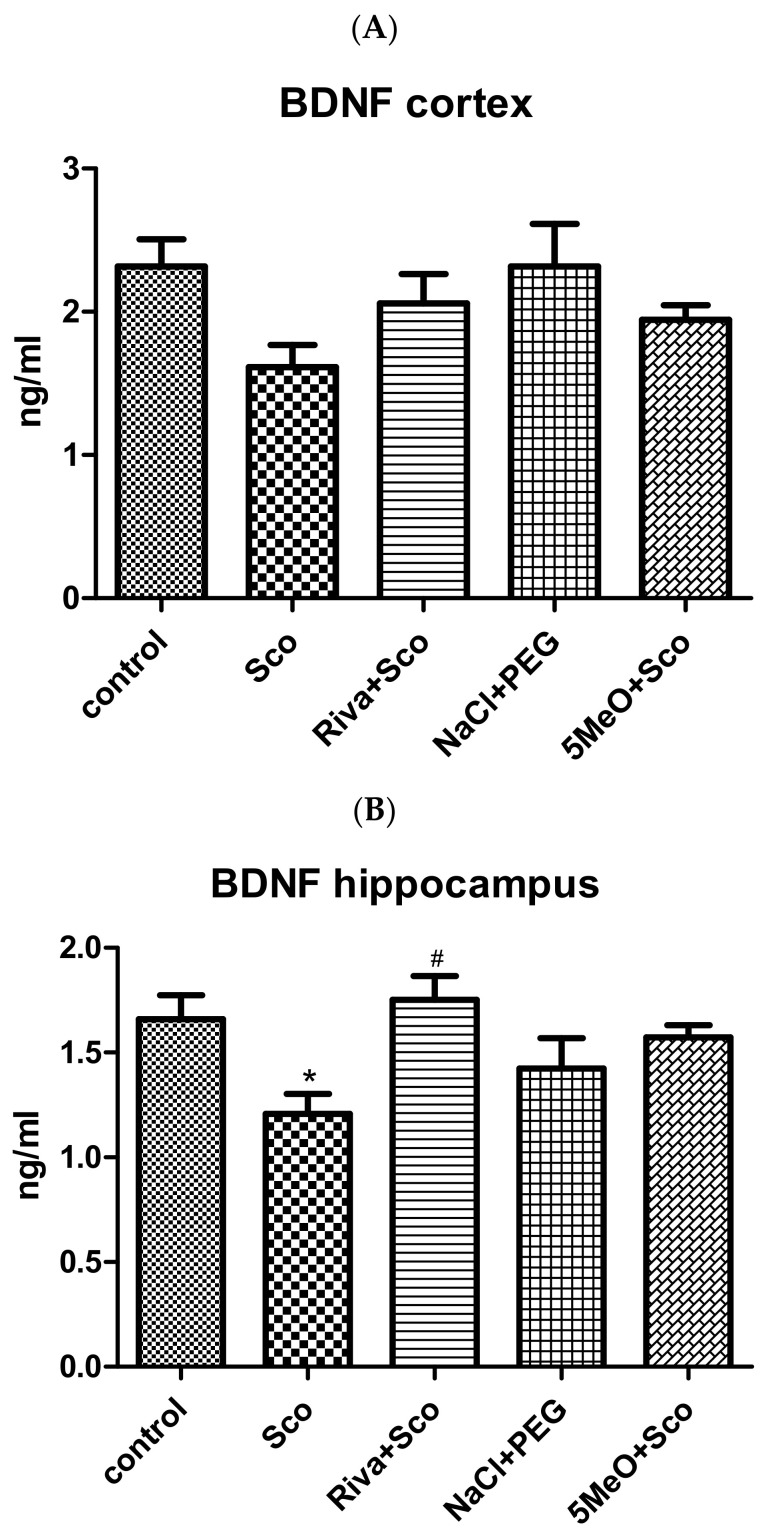
Effects of 5MeO (14.7 μM) on BDNF levels in cortex and hippocampus (**A**,**B**) of scopolamine-treated rats. Data are expressed as mean ± SEM; significant differences among experimental groups are denoted with “*” when the differences are with the saline (control) group and denoted with “#” when the differences are with the dementia (Sco) group. The number of “*” or “#” above bars is assigned as follows: # or * at *p* < 0.05.

Scopolamine decreased BDNF levels both in the cortex and hippocampus, the decrease reaching statistical significance in hippocampus (control vs. Sco, *p* < 0.05) (Figure 7A,B). In the cortex, there was no significant difference between the control and scopolamine groups nor between the control and any of the three other groups (Riva + Sco; 5MeO + Sco; NaCl + PEG). In the hippocampus, the only statistically significant difference, except between the control and scopolamine groups, was the difference between the Sco and Riva + Sco groups (Sco vs. Riva + Sco, *p* < 0.05).

### 2.3. Biochemical Evaluation of Toxicity of 5MeO (ALAT, ASAT, Creatinine, Urea, Albumin, Globulin, and Total Protein Assays)

**Figure 8:**
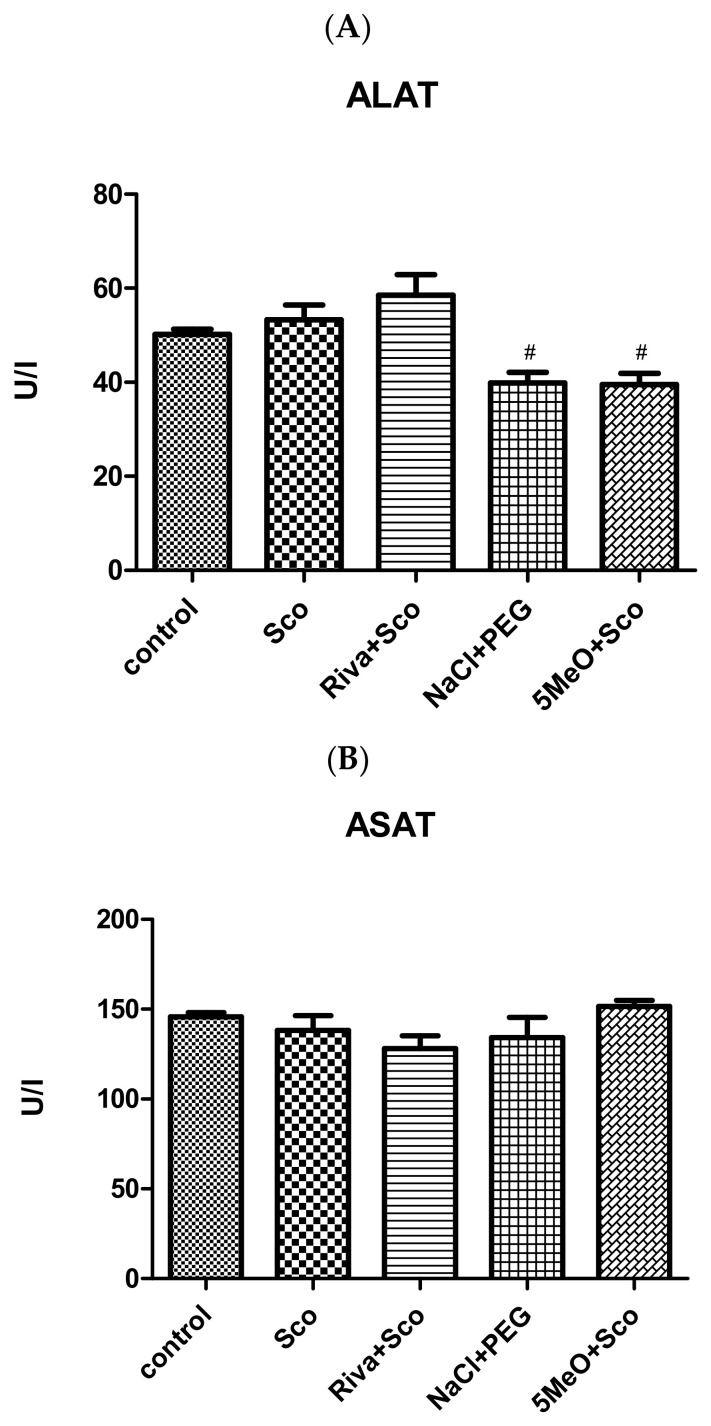

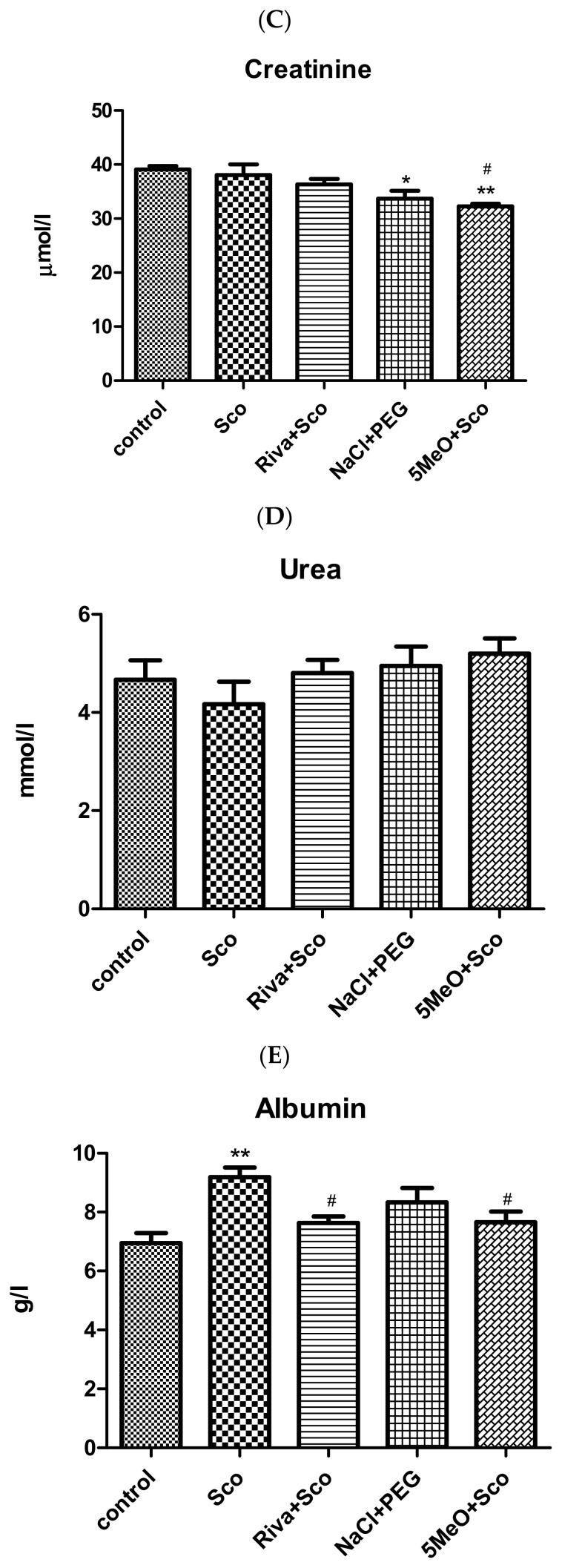

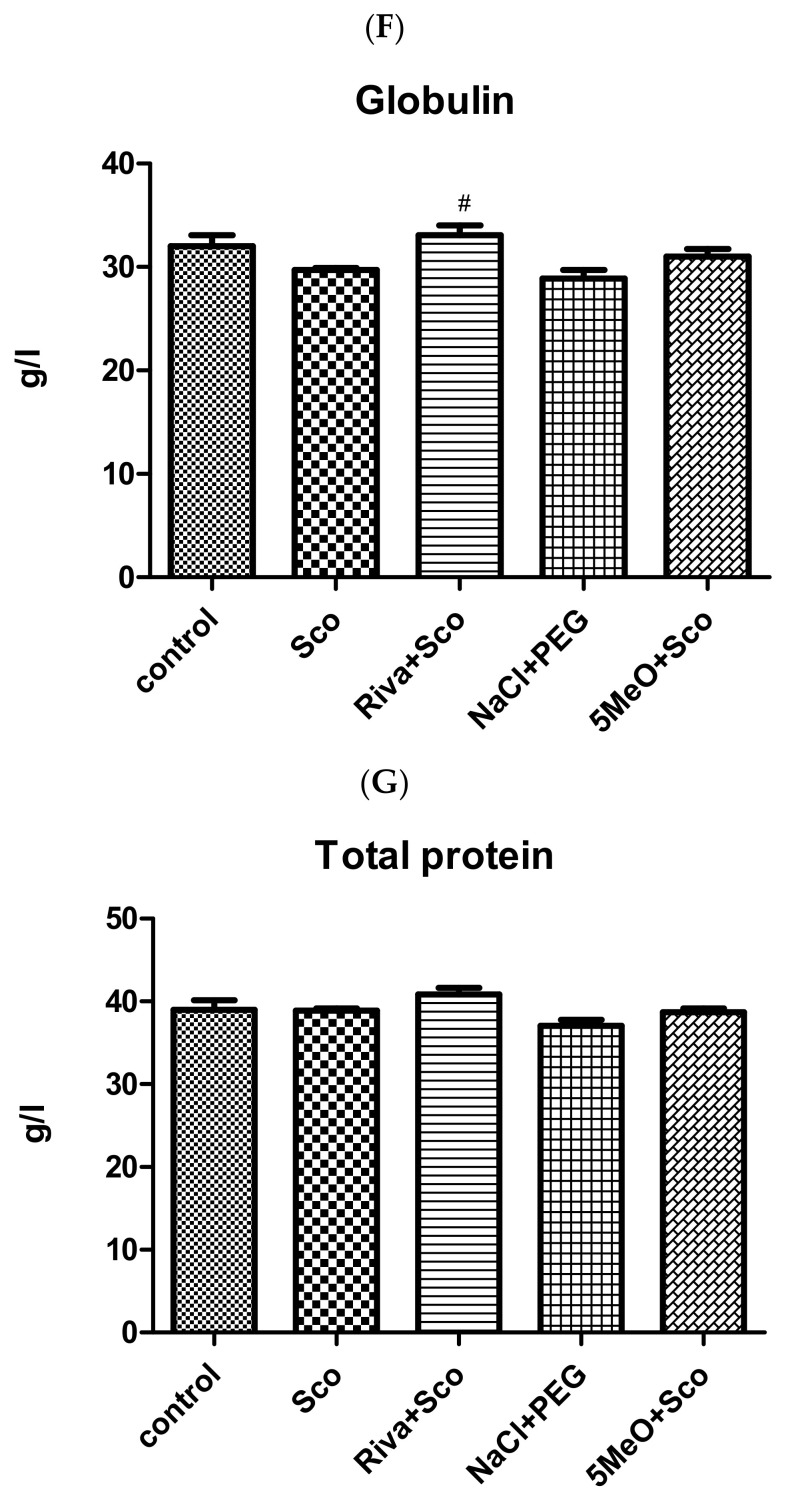
Effects of 5MeO (14.7 μM) on ASAT (**A**) and ALAT (**B**) activity and creatinine (**C**), urea (**D**), albumin (**E**), globulin (**F**), and total protein (**G**) levels of scopolamine-treated rats. Data are expressed as mean ± SEM; significant differences among experimental groups are denoted with “*” when the differences are with the saline (control) group and denoted with “#” when the differences are with the dementia (Sco) group. The number of “*” or “#” above bars is assigned as follows: # or * at *p* < 0.05; ** at *p* < 0.01.

Figure 8 shows the effects of scopolamine and 5MeO on ASAT and ALAT activity and creatinine, urea, albumin, globulin, and total protein levels. No differences between any of the groups were observed for ASAT activity and for the urea and total protein levels (Figure 8B,D,G, respectively). For ALAT activity, a significant decrease was observed for the NaCl + PEG and 5Meo + Sco groups only when compared to the scopolamine group (Figure 8A; Sco vs. NaCl + PEG, *p* < 0.05; Sco vs. 5MeO + Sco, *p* < 0.05). Similarly, no other significant changes were observed for globulin, but an increase in its levels in the Riva + Sco group compared to the rats injected only with scopolamine (Figure 8F; Sco vs. Riva + Sco, *p* < 0.05). Regarding creatinine (Figure 8C), a significant decrease in its levels, compared to the control group was observed for the animals treated with PEG and the animals treated with 5MeO in the presence of scopolamine (control vs. NaCl + PEG, *p* < 0.05; control vs. 5MeO + Sco, *p* < 0.01). A significant decrease in creatinine levels was observed in the 5MeO + Sco group also compared to the scopolamine group (Sco vs. 5MeO + Sco, *p* < 0.05). Regarding albumin (Figure 8E), scopolamine significantly increased its levels compared to the control group (control vs. Sco, *p* < 0.01), an increase that was counteracted by rivastigmine as well as 5 MeO, and there was no difference between the control and either the Riva +Sco or 5MeO + Sco groups, but there was a significant decrease in albumin levels between each of the two groups and the scopolamine group (Sco vs. Riva + Sco, *p* < 0.05; Sco vs. 5MeO + Sco, *p* < 0.05).

Compared to the control group, scopolamine induced changes only in albumin, increasing its levels, a change efficiently counteracted by both rivastigmine and 5MeO, as the two groups (Riva + Sco and 5MeO + Sco) showed no significant difference compared to the control group and a significant reduction in albumin levels compared to the scopolamine group (Sco vs. Riva + Sco, *p* < 0.05; Sco vs. 5MeO + Sco, *p* < 0.05). A difference compared to the control group was also registered for blood serum creatinine, the levels of which were significantly decreased by PEG and 5Meo in the presence of scopolamine (control vs. NaCl + PEG, *p* < 0.05; control vs. 5MeO + Sco, *p* < 0.01). The blood levels of creatinine in the 5MeO + Sco group were also decreased compared to the animals injected only with Sco (Sco vs. 5MeO + Sco, *p* < 0.05).

## 3. Discussion

In the present study, using behavioral and biochemical methods, we examined the effects on learning and different types of memory of an arylhydrazone of 5-methoxy-indole-2-carboxylic acid containing a 2,3-dihydroxy functional group (5MeO) in a model of scopolamine-induced Alzheimer-type dementia in rats. We further explored the mechanisms of those effects as well as the toxicity and safety of 5MeO. As a positive control, we utilized rivastigmine. Rivastigmine (marketed under different brand names including Exelon™, Rivastach^®^ Patch, Prometax^®^, and SDZ ENA 713) is a reversible inhibitor of both the acetylcholinesterase and butyrylcholinesterase enzymes [27] and is highly effective in the treatment of cognitive decline and Alzheimer’s disease across its mild and moderate stages, as well as in the treatment of various types of dementia including dementia associated with Parkinson’s disease [28,29,30,31,32,33].

Scopolamine is a non-selective muscarinic cholinergic receptor (mACh Rs) antagonist, known to induce deficiencies in memory and cognition elicited by dysfunction of the brain’s cholinergic system, enhanced oxidative stress and impaired antioxidants defense, neuroinflammation, unfavorable changes in the monoaminergic neurotransmitter systems, mitochondrial alterations, apoptosis, and compromised BDNF signaling [34], pathological changes accompanying AD as well [35,36,37,38,39,40,41,42,43]. Moreover, scopolamine contributes directly to the AD pathology by increasing Aβ levels and deposition (possibly due also to augmented β-site amyloid precursor protein cleaving enzyme 1 (BACE1) expression and activity [44] and the amount of phosphorylated tau protein [44,45,46,47]. Interestingly, removing the muscarinic M1 receptor in the 3xTgAD and Tg-SwDI mice (two models relevant to AD) by crossbreeding them with M(1)R(−/−) mice worsened their cognitive impairments and elevated plaque and tangle levels in the brains of the 3xTgAD mice and enhanced the cerebrovascular deposition of fibrillar Aβ in the Tg-SwDI mice [48], substantiating the role of disturbed M1 receptors (deletion or antagonism) in increasing Aβ production and exacerbating the AD pathology. Therefore, we considered the scopolamine model the appropriate tool to assess the usefulness of 5MeO as a possible candidate in treating dementia and Alzheimer’s disease.

Step-through inhibitory avoidance and the Barnes maze were the behavioral methods used to evaluate the changes in learning and memory induced by scopolamine and the capability of 5MeO to counteract them.

Step-through inhibitory avoidance is one of the most broadly used behavioral methods to study learning and memory. The step-through inhibitory avoidance task is a complex task with an emotional and spatial memory component to it and is very much dependent on the activation of the cholinergic system through the binding of acetylcholine released from cholinergic neurons originating in the basal forebrain to muscarinic cholinergic receptors in the hippocampus [49,50]. The Barnes maze task, in turn, being a classical method used to evaluate spatial learning and memory [51], is also reliant on an intact hippocampus [52,53,54,55], proper functioning of the cholinergic system [56,57], and considerably, on the activation of the hippocampal M1 muscarinic cholinergic receptors [58]. Thus, not surprisingly, as a M1 muscarinic cholinergic receptor antagonist, though not specific, and in line with the scientific literature showing scopolamine-induced worsening of the step-through and Barnes maze performances [58,59,60,61,62] in our experiments, scopolamine impaired the acquisition of both the step-through and the Barnes maze tasks by decreasing the step-through latency at the 24th hour and increasing the number of head dips in the Barnes maze compared to control animals. In the step-through test, 5MeO completely restored the STL values of scopolamine-treated rats (to that of control rats) and was equally efficient in the Barnes maze test, decreasing the number of head dips to that of control animals and showing even better results than rivastigmine.

Unravelling the mechanisms of the favorable influence on memory of 5MeO, we considered its effects on the cholinergic system by evaluating the levels of ACh and AChE activity as well as on parameters of oxidative stress by evaluating LPO and the activities of SOD, catalase, and GPx in the cortex and hippocampus.

ACh plays a fundamental role in memory formation and cognition, being central to essential processes such as attention [63,64], working memory [65,66], spatial learning and memory (for extensive review see [66,67], episodic memory [68,69], perceptual memory [70], cognitive flexibility [71], and others. AChE, being the major enzyme degrading ACh, is crucial for proper cholinergic transmission and numerous studies have demonstrated that its inhibition improves memory and slows cognitive decline in patients with various types of dementia and in AD [31,72,73]. Furthermore, accumulating evidence shows that AChE has an additional non-cholinergic effect that could exacerbate AD pathology, and especially, that AChE has been discovered to colocalize with Aβ deposits in the brains of aged people and AD patients [74]. Thus, it has been found that AChE accelerates the aggregation of Aβ in solution and that, in neuronal cultures, the AChE-Aβ complex presents itself with a stronger cytotoxic effect than Aβ fibrils alone [75,76,77]. Additionally, in hippocampal cultures, AChE-Aβ complexes provoke sustained increases in intracellular Ca^2+^ and the loss of mitochondrial membrane potential potentiating the neurodegenerative changes induced by the Aβ peptide [78]. In turn, Aβ increases AChE availability and activity by decreasing its degradation and increasing its catalytic efficiency [79,80]. In agreement with other scientific reports [47,81], our results showed that scopolamine decreases ACh levels and increases AChE activity in cortex and significantly in the hippocampus. Of particular importance is the observed significance of those changes in the hippocampus, as this brain region is vital for memory processes [82,83,84]. 5Meo showed a twofold beneficial effect on the cholinergic system by increasing ACh levels as well as decreasing AChE activity in the hippocampus and restoring them to the control levels in the scopolamine-treated group.

Oxidative stress, occurring whenever the production of reactive oxygen species exceeds the ability of the organism’s defense system to disarm them, and manifested, amongst all, by increased LPO and decreased activity of SOD, catalase, and GPx, has been proven to underlie memory impairments and cognitive dysfunction and to be a characteristic feature of neurodegeneration and AD [85,86,87,88]. Thus, in addition to its positive effects on the cholinergic function, the beneficial effects of 5MeO on memory could well be explained with its effects counteracting oxidative stress. Standing out were the effects of 5MeO on LPO levels and catalase activity. In agreement with studies both in mice and rats [89,90,91,92], our results showed that scopolamine increases MDA levels and decreases catalase activity. Although 5MeO decreased MDA levels to control levels also in the hippocampus, most conspicuous were its effects in the cortex. In the cortex, 5MeO decreased substantially the significantly increased by scopolamine MDA levels, bringing them down to the control levels, which were, at the same time, much lower than those in the presence of scopolamine alone. Such an effect was even more noticeable at the background of the effect of rivastigmine, which not only failed to decrease the significantly increased by scopolamine MDA levels but practically behaved in a manner almost identical to that of scopolamine itself. Regarding catalase, in the hippocampus (in the cortex, no significant difference between any of the groups was observed), once again, 5MeO showed better effects than rivastigmine, reflected in a significant increase in catalase activity in the 5MeO group compared to the scopolamine group of rats and no such increase in the rivastigmine group compared to the scopolamine group. The above results are very much consistent with the previous results of ours in in vitro systems, namely, the neuroprotective effects of 5MeO against H_2_O_2_-induced oxidative stress in human neuroblastoma SH-SY5Y cells and against Fe(II)-induced lipid peroxidation. Indeed, as catalase is the enzyme catalyzing the decomposition of H_2_O_2_ to water and oxygen [93], the observed in vivo enhancement of scopolamine-decreased catalase activity by 5MeO closely matches the beneficial effect of the substance in neuroblastoma cells. Those results are supported by the effects of 5MeO on GPx activity (well established is that one of the biochemical functions of GPx is to catalyse the reduction of H_2_O_2_ to water [94], which, in hippocampus, in the presence of 5MeO and scopolamine, shows no difference compared to the control GPx activity, whereas, in the presence of rivastigmine and scopolamine, it is significantly reduced. Curiously, although, in our previous studies on the scavenging activity of the superoxide anion (O^2−•^), 5MeO showed the best results from a series of similar compounds, it did not show convincing effects in vivo, failing to restore the scopolamine-decreased SOD activity in the cortex and hippocampus (SOD is known to facilitate the transition of O^2−•^ into O_2_ and H_2_O_2_ [95], a discrepancy that could be explained with a possible direct chelating or modifying effect in the solution related to the chemical structure of 5MeO against a more complex influence of the compound on the protein structure of SOD. In fact, rivastigmine showed equally bad results and, in the hippocampus, ones even worse than those of 5MeO. Interestingly, but not unexpectedly, it is on the cholinergic function that rivastigmine shows better results than 5MeO, whereas 5MeO performs better than rivastigmine fighting oxidative stress. The antioxidant capability of 5MeO is further confirmed in assays of iron-induced oxidative damage on deoxyribose where the compound suppressed deoxyribose degradation and in a model of 6-hydroxydopamine-induced oxidative stress in isolated brain synaptosomes where it demonstrated significant neuroprotection [22].

Brain-derived neurotrophic factor (BDNF) is an important neurotrophin, which in addition to neuron-supporting functions such as involvement in neuronal survival and growth and differentiation [96,97,98], has a key role in synaptic plasticity and, thus, in learning and memory [99]. The latter is further supported by studies in animals showing that genetically or pharmacologically induced deficiencies in BDNF or its receptor, tyrosine receptor kinase B (TrkB), cause memory and learning impairments [100,101] and a study in humans showing that functional loss of a copy of the BDNF gene has been linked to hyperactivity, severe obesity, and impaired cognitive function [102]. Accumulating evidence links BDNF to AD as well. It has been found that BDNF levels are decreased in the serum [103,104,105,106], cerebrospinal fluid [107], and postmortem brains of AD patients [108,109]. At the same time, higher serum BDNF levels in cognitively healthy adults lower the risk for dementia and AD [110]. The above findings parallel abundant studies in animals showing that BDNF and activating BDNF/TrkB signaling may shift the cleavage of the amyloid β precursor protein (APP) to the non-amyloidogenic pathway by stimulating α-secretase, reducing toxic Aβ generation, and exerting protective effects against Aβ-induced toxicity [111,112,113,114], whereas the interruption of BDNF signaling in hippocampal neurons activates the amyloidogenic pathway, accompanied by progressive accumulation of intra- and extracellular Aβ aggregates, and neuronal apoptotic death [115]. Moreover, data imply favorable effects of BDNF on counteracting the hyperphosphorylated tau pathology in AD. Thus, Elliot and colleagues reveal the rapid dephosphorylation of tau by BDNF dependent on TrkB activation and the downstream activation of the PI3K-Akt pathway, as well as the glycogen synthetase kinase 3β (GSK3β) [116], and Wang and colleagues demonstrate that the deprivation of BDNF/TrkB signaling increases inflammation and activates the JAK2/STAT3 pathway, resulting in an increased expression of δ-secretase, an asparagine endopeptidase, implicated in Aβ production and NFT formation [117]. Additionally, TrkB staining is inversely correlated with phosphorylated tau staining in the hippocampus of AD patients [118]. All aforementioned findings suggest that increasing BDNF levels, including in regions important for complex learning and memory such as the hippocampus, may relieve cognitive deficits and AD pathology, slow neurodegeneration and improve clinical outcome for patients. Scopolamine decreases BDNF levels in the hippocampus, [119,120,121] which matches our results showing a significant reduction in the neurotrophin in the brain region under the influence of the muscarinic receptor antagonist. 5MeO increases BDNF levels in the scopolamine-treated group, restoring them to control values, another quality of 5MeO that we believe accounts for its memory-consolidating effect in the Barnes maze and the passive avoidance tasks, whose proper accomplishment is dependent on activation of BDNF/TrkB signaling [100,122].

In addition to studying the effects of 5MeO on learning and different types of memory and examining the mechanisms of these effects through studies on AChE activity, ACh levels, oxidative stress, and antioxidant defense systems markers as well as BDNF, we evaluated its toxicity and safety of use. The examination of blood biochemical parameters such as ALAT, ASAT, creatinine, urea, albumin, globulin, and total protein showed the lack of side effects on liver and kidney function and demonstrated the safety of the use of 5MeO. Of the many parameters tested, scopolamine induced changes only in albumin by increasing its levels, a change that was effectively counteracted by 5MeO since the group of rats injected with 5MeO and scopolamine showed no significant difference compared to the control group and a significant decrease in albumin levels compared to the scopolamine group. Regarding blood serum creatinine, its levels were significantly decreased in the group treated simultaneously with 5MeO and scopolamine compared to the control group, but the values still remained within the reference range for rats [123,124]. Our in vivo studies demonstrating lack of toxicity of 5MeO are in line with our previous in vitro results [22]. The SH-SY5Y human neuroblastoma cell line is a relevant in vitro cell model for investigating neurotoxicity in humans [125] and our results showed that, based on assessment of cell viability, 5MeO did not exhibit any significant cytotoxic effects (IC_50_ = 111 μM) [22]. It is possible that the damaging effects of 5MeO could extend further than cytotoxicity, leading to disruption of the blood–brain barrier (BBB) (shown to be linked to neuroinflammation and neurodegenerative diseases [126,127,128]) by affecting the tight junctions between endothelial cells of the cerebral vasculature resulting in bypassing transcellular in favor of paracellular transport. Our previous studies on immortalized mouse brain microvascular endothelial cells, the bEnd3 cell line, an ascertained and convenient model for evaluating BBB function [129], showed permeability for 5MeO of the bEnd3 cell monolayer without affecting zonula occludens-1 (ZO-1) and the tight junctions (TJ) intactness [22]. Additionally, the lack of red blood cells hemolysis induced by 5MeO (below 5% even at the highest tested concentration of 200 µM) and the low toxicity on freshly isolated brain synaptosomes [22] further characterize 5MeO, completing its favorable safety profile.

## 4. Conclusions

Given the complex pathology of AD, where oxidative stress, neuroinflammation, and amyloid-tau dysfunction converge, 5MeO presents a compelling candidate for further pharmacological studies. Previously explored for Parkinson’s disease in in vitro model systems, 5MeO demonstrated potent neuroprotective effects, strong antioxidant properties, and significant inhibition of MAO-B, a key enzyme implicated in both Parkinson’s and Alzheimer’s disease. Our present experiments, in vivo, in a model of scopolamine-induced Alzheimer-type dementia in rats, demonstrated that 5MeO had a beneficial effect on different types of memory as assessed by the step-through and the Barnes maze tasks. It efficiently restored the decreased by scopolamine BDNF and ACh levels and the increased by scopolamine AChE activity in hippocampus. Most effective 5MeO was in combating the induced by scopolamine oxidative stress by reducing the levels of lipid peroxidation and by increasing catalase activity. The multitude of the effects of 5MeO, together with its favorable safety profile and permeability across the blood–brain barrier, imply therapeutic benefits beyond symptomatic relief and suggest 5MeO as a successful tool in a multitargeted treatment of AD.

## 5. Materials and Methods

### 5.1. Synthesis

The synthesis, the structural data (IR, ^1^H, ^13^C NMR, mass spectroscopy), and the crystal structure of the target compound have been published elsewhere [22,130].

### 5.2. Laboratory Animals

The experiments were conducted on male adult Wistar rats (180–260 g), bred in Experimental Breeding Facility, Slivnitsa, Sofia. The animals were transported to the Institute of Neurobiology, Bulgarian Academy of Sciences, housed in plastic cages (*n* = 5), with free access to water and food and habituated to the conditions of the local vivarium (standard laboratory conditions such as 12 h light/dark cycle and ventilation of the rooms providing optimal temperature and humidity).

All experimental procedures were conducted in agreement with the guidelines of the Bioethics Committee at the Institute of Neurobiology, Bulgarian Academy of Sciences, Bulgaria in compliance with the national and international laws and policies (the new Directive 2010/63/EU (22 September 2010) of the European Parliament and the Council of the European Union replacing the older Council Directive (86/609/EEC) on the protection of animals used for scientific purposes; the National Institute of Health (NIH) Guide for the Care and Use of Laboratory animals, NIH Publication No. 85-23, 1985; and Bulgarian laws (Ordinance No. 20 of 1 November 2012 on the minimum requirements for the protection and welfare of experimental animals and the facilities for their use, breeding and/or supply, issued by the Ministry of Food and Agriculture, Official Gazette No. 87 of 9 November 2012 and in effect from 1 January 2013.

### 5.3. Experimental Groups and Design

The animals were distributed into experimental groups, each with 8 rats, as follows:

- Control group (control)—receiving saline (0.5 mL/100 g, i.p.) for 11 days;
- Scopolamine group (Sco)—receiving 2 mg/kg scopolamine (0.5 mL/100 g, i.p.) for 11 days;
- Rivastigmine and scopolamine group (Riva + Sco)—receiving 2.5 mg/kg Rivastigmine (0.5 mL/100 g, i.p) and 2 mg/kg scopolamine (0.5 mL/100 g, i.p.) for 11 days;
- Polyethylene glycol group (NaCl + PEG)—receiving Polyethylene glycol (30%) in 0.9% NaCl (0.5 mL/100 g, i.p.) for 11 days;
- N′-(3,4-dihydroxybenzylidene)-5-methoxy-1H-indole-2-carbohydrazide) (5MeO) and scopolamine group (5MeO + Sco)—receiving 14.7 μM (4.86 mg/mL) 5MeO in 30% PEG in 0.9% NaC (0.5 mL/100 g, i.p.) and 2 mg/kg scopolamine (0.5 mL/100 g, i.p.) for 11 days.

**Scheme 2:**
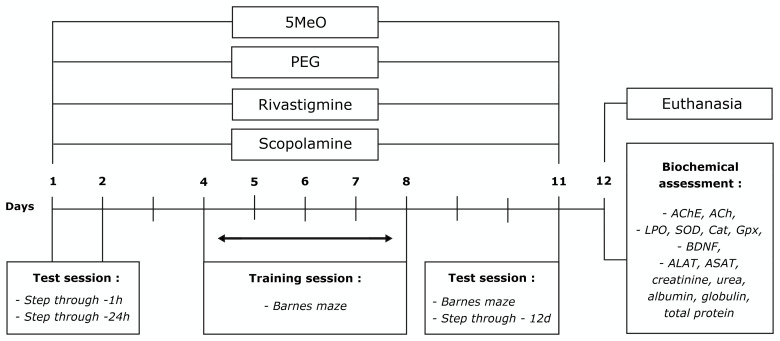
Diagram of experimental design.

Evaluation of behavioral and biochemical changes induced by 5MeO, rivastigmine, and PEG was performed using a model of Alzheimer’s-type dementia in rats. Based on previously conducted studies [131], experimental dementia of Alzheimer’s type was induced chemically in laboratory male Wistar rats by daily administrations of scopolamine hydrobromide (11 days, 2.0 mg/kg intraperitoneally). On the 11th day of treatment, behavioral tests such as step-through inhibitory avoidance and the Barnes maze tasks were performed, and on the 12th day after the start of the treatment, the rats were euthanized under CO_2_ inhalation and sacrificed for biochemical studies (Scheme 2). Blood was used for assessment of parameters of toxicity such as alanine aminotransferase (ALAT) and aspartate transferase (ASAT) activities, creatinine, and urea levels, as well as total protein, albumin, and globulin levels, and brains (cortex and hippocampus, separately) were used for assessment of acetylcholinesterase (AChE) activity, acetylcholine (ACh) levels, and lipid peroxidation levels (LPO) as judged by the level of malondialdehyde (MDA), superoxide dismutase (SOD) activity, catalase activity, glutathione peroxidase (GPx) activity, and brain-derived neurotrophic factor (BDNF) levels.

### 5.4. Behavioral Experimental Methods

#### 5.4.1. Step-Through Inhibitory (Passive) Avoidance

Step-through inhibitory (passive) avoidance is one of the most frequently employed methods for evaluation of learning and memory in experimental animals as it is simple, yet informative, allowing study of recall in a one-trial session, at varied times after acquisition [132].

The equipment is divided into two compartments, one lighted and the other dark, parted by a guillotine door. The floor of the dark compartment is covered with a still grid for delivering electric shocks once the rodent has stepped in with all four paws. The experiment is accomplished in two sessions, a training and a test session [133]. During the training session, which proceeds the beginning of scopolamine administrations, rats are positioned in the lighted compartment for a period of habituation (1 min), after which the guillotine door is opened, allowing rats access to the dark compartment, upon entry of which they receive an unavoidable electric shock (0.7 mA, 3 s). During the test session, carried out at the 1st and 24th hour and on the 12th day after the start of scopolamine administrations, rats are again positioned in the lighted compartment and once again allowed access to the dark compartment but without receiving an electric shock. The time necessary for the animals to enter the dark compartment is recorded as step-through latency (STL) (cut-off time 180 s) and used to assess impairments in learning and memory in the various experimental groups.

#### 5.4.2. Barnes Maze

The Barnes maze is ordinarily used to determine changes in spatial learning and memory in rodents [133]. It uses a circular platform (122 cm in diameter, 80 cm above the ground) with 20 peripheral holes and a dark box under one of them serving as an escape. Once the animal is placed in the middle of the open platform a stimulus of strong light (3000 lx) and loud noise (76 dB) forces it to seek refuge and hide in the escape box. Assessment of spatial memory is achieved by comparing the times it takes the animals to find and enter the hole with the escape box below (total latency) and the number of “error” holes peeks before reaching it (denoted as “number of head dips”). The experiment is conducted in two sessions, a “training” and a “test” session, according, with minor alterations, to a previously reported protocol [131]. The “training” session was performed on the 4th day after the beginning of scopolamine treatment, and the “test session” on the 12th day after the first administration of scopolamine. Training was carried out on 4 consecutive days. On the first day, over 5 min, the animals were allowed to freely explore the platform. During the next 3 days, each day, 4 trials (separated by 15 min) were conducted for each animal with the animal initially positioned in the center of the platform and let free to search the escape box. The time needed by the animal to find the hole with the escape box underneath and the number of “error” holes before finding it were recorded. On the first of the 3 days, the experiment was carried out without the repulsive stimulus, and the next two days with the light and noise on. The test session was conducted in a way similar to on the last day training, namely, 4 trials (separated by 15 min) for each animal, recording the time to find the hole with the escape box underneath and the number of “incorrect” holes examined.

### 5.5. Biochemical Methods

Tissue preparation: On the day following behavioral experiments, the rats were euthanized and decapitated and their brains were quickly removed. The main brain structures related to learning and memory, the cortex and hippocampus, were carefully extracted on ice and prepared for biochemical analyses. After homogenization with an ultrasonic homogenizer in PBS (0.01 M, pH 7.4) (tissue (g): PBS (mL), 1:9), the tissues were centrifuged for 5 min at 5000× *g*. Part of the obtained post-nuclear supernatant fraction was used for subsequent spectrophotometrical measurement of levels of lipid peroxidation (LPO) and acetylcholine (ACh), acetylcholinesterase (AChE) activity and the content of brain-derived neurotrophic factor (BDNF). The remaining part of the post-nuclear supernatant fraction was further centrifuged for 20 min at 12,000× *g* and the new supernatant used for measurement of superoxide dismutase (SOD), catalase and glutathione peroxidase (GPx) activities. All manipulations were performed between 0° and +4 °C.

AChE activity in the cortex and hippocampus was measured using the Ellman’s method [134]. The Ellman’s method follows the increase in the yellow color of thiocholine produced by the catalytic action of AChE when 5,5′-dithio-bis(2-nitrobenzoic acid (DTNB) is added to solution. We mixed 100 µL of the supernatant with the Ellman reagent of the following composition: 2.9 mL 0.1 M phosphate buffer (pH 8), 100 µL 0.1 M DTNB, and 20 µL 0.075 M freshly prepared acetylthiocholine iodide. Then, 500 µL of the above reaction mixture was injected into a Semi-auto Chemistry Analyzer and the kinetics of the reaction were monitored at 405 nm for 3 min.

ACh levels in the cortex and hippocampus were measured using a commercially available sandwich ELISA kit (MyBioSource, Inc., San Diego, CA, USA, Catalog No MBS164787). Briefly, 50 µL of standards (12.5–200 U/mL) were added to wells assigned for standards and 40 µL sample + 10 µL anti-ACh antibody was added to wells assigned for samples. Next, 50 µL streptavidin-Horseradish Peroxidase (HRP) was added to sample as well as standard wells and the plate was covered and incubated for 60 min at 37° in the dark. After the incubation the liquid was removed and all wells washed 5 times with a wash buffer. At a next step 50 µL substrate solution A and 50 µL substrate solution B were added to each well of the plate and the plate was further incubated for 10 min at 37° in the dark. The color reaction was stopped with 50 µL Stop solution, followed by the immediate recording of the absorbance at 450 nm (MR-96A microplate reader).

Oxidative stress parameters were determined using Sigma-Aldrich Co. LLC, Louis, MI, USA kits as follows:

Lipid peroxidation levels in cortex and hippocampus were determined using assay kit MAK085 and expressed as nmoles malondialdehyde (MDA)/mg protein, using a molar extinction coefficient of 1.56 × 106 M^−1^ cm^−1^.

Superoxide dismutase activity in cortex and hippocampus was determined using assay kit WST 19160 and expressed as units/mg protein, one unit being the amount of enzyme needed to inhibit the water-soluble tetrazolium (WST) reduction by 50%.

Catalase activity in the cortex and hippocampus was determined using assay kit CAT100 and expressed as units/mg protein, corresponding to ΔA240/min/mg protein, ΔA240 being the absorption decrease at 240 nm related to the decomposition of H_2_O_2_ under the catalytic action of catalase.

Glutathione peroxidase activity in the cortex and hippocampus was determined using assay kit Glutathione Peroxidase Cellular Activity Assay CGP1 and expressed as units/mg protein relying on a molar extinction coefficient of 6.22 mM^−1^cm^−1^ for NADPH.

Protein concentrations were determined according to the Lowry method [135] with the curve used for calculations based on bovine serum albumin as a standard.

BDNF levels in cortex and hippocampus were determined using a standard sandwich ELISA kit (Catalog No. MBS457896, MyBioSource, Inc., San Diego, CA, USA). In brief, 100 µL of BDNF standards (0.156–10 ng/mL) and 100 µL of BDNF samples were added to the wells of the microplate, each of which pre-coated with BDNF. Next, the plate was incubated in the absence of light for 90 min at 37°. In a next step, after the liquid from each well was removed, 100 µL Detection Solution A were added, and the plate was incubated again for 45 min at 37°. After the incubation, a wash step included washing each well 3 times with a washing buffer. The same procedure, as with Detection solution A, was repeated with Detection Solution B, followed by washing. The addition of 3,3′,5,5′-tetramethylbenzidine (TMB) gave a start to a color reaction, stopped 10 min later with a Stop solution, followed by the immediate recording with a MR-96A microplate reader of the absorbance at 450 nm.

To measure alanine aminotransferase (ALAT) and aspartate transferase (ASAT) activities, creatinine, and urea levels, as well as total protein, albumin, and globulin levels, blood from the experimental animals was collected in serum separator tubes and centrifuged for 10 min at 3000 rpm to obtain serum. Following instructions in the supplied protocols, commercially produced standard kits by Giesse Diagnostics kit, Italy were used for determining all the parameters (ALAT (Catalog No. 419407); ASAT (Catalog No. 419107); creatinine (Catalog No. 006007); urea (Catalog No. 4164/B); total protein (Catalog No. E020)) except albumin, the levels of which were determined by a kit by BioSino Bio-Technology and Science Inc., Beijing, China (catalog No. E019). Globulins levels were obtained by subtracting albumin levels from the levels of total protein.

### 5.6. Statistical Analysis

Results are presented as mean values ± SEM. Statistical analysis of the parameters is performed using GraphPad Prism 5.0 and one-way analysis of variance (ANOVA) followed by the Tukey’s multiple comparison post hoc test. Differences were considered significant at *p* < 0.05.

## Data Availability

The original data presented in the study are included in the article; further inquiries can be directed to the corresponding authors.
